# Impact of Web Searching and Social Feedback on Consumer Decision Making: A Prospective Online Experiment

**DOI:** 10.2196/jmir.963

**Published:** 2008-01-22

**Authors:** Annie YS Lau, Enrico W Coiera

**Affiliations:** Centre for Health InformaticsUniversity of New South WalesSydneyAustralia

**Keywords:** Health care consumer, information searching, decision making, social feedback, Internet, accuracy, confidence

## Abstract

**Background:**

The World Wide Web has increasingly become an important source of information in health care consumer decision making. However, little is known about whether searching online resources actually improves consumers’ understanding of health issues.

**Objectives:**

The aim was to study whether searching on the World Wide Web improves consumers’ accuracy in answering health questions and whether consumers’ understanding of health issues is subject to further change under social feedback.

**Methods:**

This was a pre/post prospective online study. A convenience sample of 227 undergraduate students was recruited from the population of the University of New South Wales. Subjects used a search engine that retrieved online documents from PubMed, MedlinePlus, and HealthInsite and answered a set of six questions (before and after use of the search engine) designed for health care consumers. They were then presented with feedback consisting of a summary of the post-search answers provided by previous subjects for the same questions and were asked to answer the questions again.

**Results:**

There was an improvement in the percentage of correct answers after searching (pre-search 61.2% vs post-search 82.0%, *P <*.001) and after feedback with other subjects’ answers (pre-feedback 82.0% vs post-feedback 85.3%, *P =*.051).The proportion of subjects with highly confident correct answers (ie, confident or very confident) and the proportion with highly confident incorrect answers significantly increased after searching (correct pre-search 61.6% vs correct post-search 95.5%, *P <*.001; incorrect pre-search 55.3% vs incorrect post-search 82.0%, *P <*.001). Subjects who were not as confident in their post-search answers were 28.5% more likely than those who were confident or very confident to change their answer after feedback with other subjects’ post-search answers (*χ* ^2^_1_= 66.65, *P <*.001).

**Conclusions:**

Searching across quality health information sources on the Web can improve consumers’ accuracy in answering health questions. However, a consumer’s confidence in an answer is not a good indicator of the answer being correct. Consumers who are not confident in their answers after searching are more likely to be influenced to change their views when provided with feedback from other consumers.

## Introduction

The World Wide Web is now recognized as an important source of information in supporting the practice of evidence-based medicine [1] and consumer health care decision making [2]. While much research focuses on the impact of information retrieval on clinical decision making, there has been little examination of how online searching influences the way consumers make health-related decisions.

Many studies have examined the quality of online health care consumer information [3], the tools and initiatives developed to promote health literacy [4], as well as the characteristics of websites and search engines that influence the way consumers perceive and utilize information [5,6]. Of particular relevance to understanding the way consumers use online health-related information, past studies have examined consumers’ familiarity with health vocabulary [7], their information appraisal [8] and search query reformulation skills [9], the way they perceive and assess Web-based health information [10-13], the types of online sources they trust [14], the patterns of use and barriers experienced while using online resources [15], and how access to online information influences the way they interact with health care professionals [16-18].

Studies have also shown that people are an important source of influence among consumers with a health-related concern. In a randomized controlled trial conducted by Lorig et al, patients with back pain who had access to an email discussion group demonstrated greater improvement in pain and made less physician visits than those without access [19]. Patients with breast cancer participating in electronic support groups are reported to have reduced rates of depression and lessened reactions to pain [20].

Little, however, is known about whether consumers are actually able to improve their understanding of health issues after searching the Web. In addition, little is known about the extent to which social feedback affects the way consumers develop their understanding of health issues. This prospective experiment tests the following hypotheses: (1) consumers can improve their accuracy in answering health care questions after searching tested online resources, and (2) consumers’answers to health care questions are influenced by feedback with other consumers’ answers.

## Methods

### Study Design

A convenience sample of 227 undergraduate students was recruited from the University of New South Wales (UNSW). Subjects were asked to use a specific online search engine to answer six consumer health questions. People with Internet access who had previously used an online search engine were recruited by announcements via student email lists, posters, leaflets, weekly student magazines, and a UNSW research news website. Upon completion of the study, subjects were entered into a draw for one of 100 movie tickets. Ethics approval was obtained from the Human Research Ethics Advisory Panel at UNSW.

**Figure 1:**
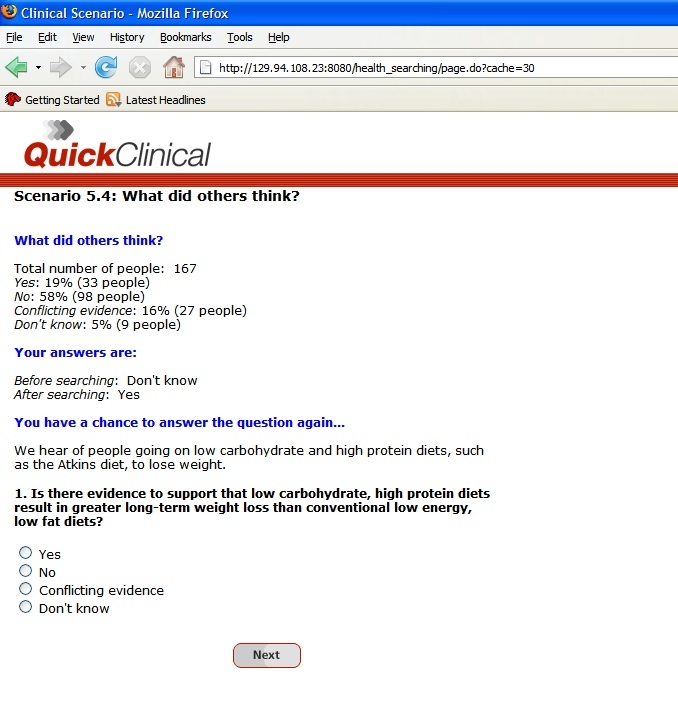
Screen capture of feedback provided to subjects after answering a question post-search

A pre/post protocol was used in this study. Subjects recorded their pre- and post-search answers to each question and their confidence in these answers. After answering each question post-search, subjects were presented with a summary of the post-search answers provided by previous subjects and were asked to answer the question again (Figure 1).

Each question and the expected correct answer are shown in Table 1. All scenario questions were randomly allocated. There were four options to answer a question: “yes,” “no,” “conflicting evidence,” and “don’t know.” Confidence was measured by a 4-point Likert scale from “very confident” to “not confident.” The questions ranged in difficulty and topic in order to cover a spectrum of health care consumer questions. They were developed in consultation with a general practitioner and two academics from the School of Public Health and Community Medicine at UNSW. Agreement was reached on the “correct” answer and the location of the best evidence sources for each question. A pilot test with three members of the general public tested the questions for interest and readability. Two additional pilots of five people each were conducted to confirm that it was possible to locate documentary evidence required to answer the questions correctly.

The search engine retrieved documents from tested resources known to have high relevance in answering health-related questions [21]. These resources are PubMed [22], MedlinePlus [23], and HealthInsite [24]. Overall, subjects were advised to spend about 10 minutes for each question and to use only the provided search system to answer the questions. To prevent subjects from visiting external websites during the experiment, the navigational bar on the Web browser was hidden once the subject logged on to the study website.

**Table 1:** Case scenarios and questions presented to subjects. A random selection of six cases was presented to each subject in the study.

**CaseScenario and Question (Scenario Name)**	**Expected Correct Answer**
1. We hear of people going on low carbohydrate and high protein diets, such as the Atkins diet, to lose weight. *Is there evidence to support that low carbohydrate, high protein diets result in greater long-term weight loss than conventional low energy, low fat diets? *(Diet)	No
2. You can catch infectious diseases such as the flu from inhaling the air into which others have sneezed or coughed, sharing a straw, or eating off someone else’s fork. The reason is because certain germs reside in saliva, as well as in other bodily fluids. Hepatitis B is an infectious disease. *Can you catch Hepatitis B from kissing on the cheek?* (Hepatitis B)	No
3. After having a few alcoholic drinks, we depend on our liver to reduce the Blood Alcohol Concentration (BAC). Drinking coffee, eating, vomiting, sleeping, or having a shower will not help reduce your BAC. *Are there different recommendations regarding safe alcohol consumption for males and females?* (Alcohol)	Yes
4. Sudden infant death syndrome (SIDS), also known as “cot death,” is the unexpected death of a baby where there is no apparent cause of death. Studies have shown that sleeping on the stomach increases a baby’s risk of SIDS. *Is there an increased risk of a baby dying from SIDS if the mother smokes during pregnancy?* (SIDS)	Yes
5. Breast cancer is one of the most common types of cancer found in women. *Is there an increased chance of developing breast cancer for women who have a family history of breast cancer? *(Breast cancer)	Yes
6. Men are encouraged by our culture to be tough. Unfortunately, many men tend to think that asking for help is a sign of weakness. *In Australia, do more men die by committing suicide than women*? (Suicide)	Yes
7. Many people use home therapies when they are sick or to keep healthy. Examples of home therapies include drinking chicken soup when sick, drinking milk before bed for a better night’s sleep, and taking vitamin C to prevent the common cold. *Is there evidence to support the taking of vitamin C supplements to help prevent the common cold?* (Cold)	No
8. We know that we can catch AIDS from bodily fluids, such as from needle sharing, having unprotected sex, and breast-feeding. We also know that some diseases can be transmitted by mosquito bites. *Is it likely that we can get AIDS from a mosquito bite?* (AIDS)	No

### Data Analysis

Subjects’ searches and their selected documents, pre-/post-search answers and confidence, post-feedback responses, time taken from answering the question pre-search to answering post-search, and responses to the pre-search and post-search questionnaire were logged during the experiment. Responses to questions were coded as “correct,” “don’t know,” or “incorrect” according to the predetermined answers for each question. All cases in which subjects did not conduct a search before providing an answer or seeking the social feedback, did not answer the question post-search, or answered “don’t know” post-search were removed from the data analysis.

The test for difference between proportions was used to compare differences between subjects’ pre-search, post-search, and post-feedback answers and to compare changes in confidence in answers pre- and post-search. The chi-square test was used to examine whether there was a statistically significant relationship between subjects’ confidence in their post-search answers and their tendency to change answers after feedback with other subjects’ answers. The McNemar test was used to examine the direction of change in pre- and post-feedback answers.

## Results

### Subjects and Sample

**Figure 2:**
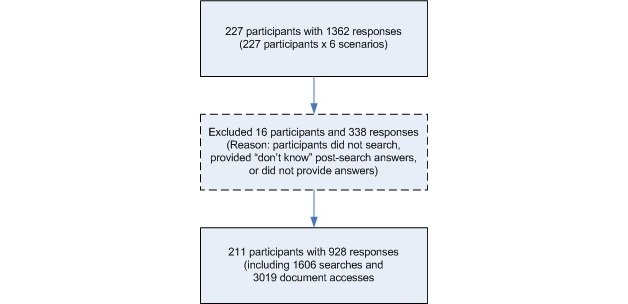
Data exclusion procedure

After data exclusion (Figure 2), the study consisted of 211 subjects who made 928 responses, 1606 searches, and 3019 document accesses. Table 2 presents demographic attributes and self-rated search skills and frequency of searching the Web for general topics and health-related issues. Overall, subjects on average took 361 seconds (SD 281.2) to search, made 1.73 (SD 1.391) searches, and accessed 3.25 (SD 3.067) documents to answer a question.

**Table 2:** Characteristics of subjects (N = 211)

**Characteristic**	**No. (%)**
**Gender**	
Female	130 (61.6)
Male	81 (38.4)
**Age (years)**	
<25	139 (65.9)
25 to 34	46 (21.8)
35 to 44	12 (5.7)
≥ 45	14 (6.6)
**Search skill**	
Fair or poor	46 (21.8)
Good	100 (47.4)
Very good	65 (30.8)
**Search frequency**	
Once a week or less	13 (6.2)
Several times a week	198 (93.8)
**Health search frequency**	
Never	9 (4.3)
Less than once a week	94 (44.5)
Once a week	52 (24.6)
Several times a week	56 (26.5)

### Impact on Decision Accuracy

As shown in Table 3, most subjects, 56.5% (95% CI: 53.3-59.6), answered correctly both before and after searching, which was termed right-right (RR). This was followed by 25.5% (95% CI: 22.8-28.4) who improved their answers after searching, wrong-right (WR), 13.3% (95% CI: 11.2-15.6) who never answered correctly, wrong-wrong (WW), and 4.7% (95% CI: 3.6-6.3) who went from right to wrong (RW).

The test for difference between proportions shows that there was a statistically significant improvement (21%) in the percentage of correct answers before and after searching(pre-search 61.2% [95% CI: 58.0-64.3]; post-search 82.0% [95% CI: 79.4-84.3]; *z*= −1.21, *P <*.001). There was also a marginal significant improvement in the percentage of correct answers before and after feedback with other subjects’ answers (pre-feedback 82.0% [95% CI: 79.4-84.3]; post-feedback 85.3% [95% CI: 82.9-87.5]; *z*= −1.95, *P =*.051; Table 4).

**Table 3:** Changes in answer before and after searching (N = 928; adapted from [25])

**Pre-Search**	**Post-Search**	**Percentage(95% CI)**	**Total No.**
Right	Right	56.5 (53.3-59.6)	524
Wrong	Right	25.5 (22.8-28.4)	237
Wrong	Wrong	13.3 (11.2-15.6)	123
Right	Wrong	4.7 (3.6-6.3)	44

**Table 4:** Correct answers by case scenario (N = 928)

**CaseScenario (n)**	**Correct Before Searching,** **No. (%)**	**CorrectAfter Searching, No. (%)**	**Correct After Feedback, No. (%)**
Diet (115)	38 (33.0)	72 (62.6)	79 (68.7)
Hepatitis B (123)	90 (73.2)	108 (87.8)	114 (92.7)
Alcohol (113)	93 (82.3)	94 (83.2)	99 (87.6)
SIDS (111)	71 (64.0)	95 (85.6)	97 (87.4)
Breast cancer (121)	108 (89.3)	108 (89.3)	111 (91.7)
Suicide (113)	63 (55.8)	98 (86.7)	104 (92.0)
Cold (111)	22 (19.8)	68 (61.3)	71 (64.0)
AIDS (121)	83 (68.6)	118 (97.5)	117 (96.7)
Total (928)	568 (61.2)	761 (82.0)	792 (85.3)

### Impact of Confidence

Table 5 shows that the most frequently self-reported change in confidence for all responses before and after searching was “increased confidence” (WW 51.9% [95% CI: 42.5-61.0], WR 54.0% [95% CI: 46.3-61.6], RW 40.4% [95% CI: 27.6-54.7], RR 71.1% [95% CI: 67.4-74.6]).

More than half of subjects (55.6%; 95% CI: 37.3-72.4) who did not know the answer pre-search and answered incorrectly post-search (DW) reported that they were confident or very confident with their incorrect post-search answer (Table 6). In fact, 82.0% (95% CI: 75.5-87.1) of subjects who were incorrect post-search reported being confident or very confident with their post-search answer (Table 7). Although Table 7 shows that the proportion of subjects with highly confident correct answers (ie, confident or very confident) significantly increased after searching (pre-search 61.6% [95% CI: 57.6-65.5]; post-search 95.5% [95% CI: 93.8-96.8]; *z*= –15.60, *P <*.001), the proportion of subjects with highly confident incorrect answers also increased after searching (pre-search 55.3% [95% CI: 50.1-60.3]; post-search 82.0% [95% CI: 75.5-87.1]; *z*= –6.75, *P <*.001).

**Table 5:** Changes in confidence in original answer following searches (N = 905; adapted from [26])^*^

**Change inConfidence**	**WW**^†^**(n = 108), No. (%)**	**WR**^†^**(n = 161), No. (%)**	**RW (n = 47), No. (%)**	**RR (n = 589), No. (%)**
Decreased	15 (13.9)	58 (36.0)	14 (29.8)	5 (0.8)
No change	37 (34.3)	16 (9.9)	14 (29.8)	165 (28.0)
Increased	56 (51.9)	87 (54.0)	19 (40.4)	419 (71.1)

^*^In 23 responses, subjects did not report a confidence rating.

^†^Includes subjects who did not know the answer before searching.

**Table 6:** Confidence in post-search answer for subjects who did not know answer before searching (N = 147; adapted from [26])

**Post-Search Confidence**	**Wrong After Search(n = 27), No. (%)**	**Right After Search(n = 120), No. (%)**
Not confident /somewhat confident	12 (44.4)	13 (1.8)
Confident /very confident	15 (55.6)	107 (89.2)

**Table 7:** Comparison of confidence between pre-search and post-search right and wrong answers (N = 928)

**Confidence inAnswer**	**Pre-Search, No. (%)**	**Post-Search, No. (%)**	***z*****Score**	***P*****Value**
**Right answer**	(n= 568)	(n = 761)		
Not confident/ somewhat confident	208 (36.6)	34 (4.5)	14.91	< .001
Confident/ very confident	350 (61.6)	727 (95.5)	–15.60	< .001
Not provided	10 (1.8)	–	–	–
**Wrong answer**	(n= 360)	(n = 167)		
Not confident/ somewhat confident	154 (42.8)	30 (18.0)	6.28	< .001
Confident/ very confident	199 (55.3)	137 (82.0)	–6.75	< .001
Not provided	7 (1.9)	–	–	–

### Impact of Social Feedback

Those who were not as confident in their post-search answers were 28.5% more likely than those who had higher levels of confidence to change their answer after feedback with other subjects’ post-search answers (not confident / somewhat confident 34.4% [95% CI: 23.9-46.6]; confident / very confident 5.9% [95% CI: 4.5-7.7]; *χ* ^2^_1_= 66.65, *P <*.001; Table 8). Those who changed their answer after feedback were more likely to change it from wrong to right than from right to wrong (McNemar *χ* ^2^_1_= 15.25, *P <*.001; Table 9).

**Table 8:** Number of subjects who changed their post-search answer after feedback (N = 928)

**Post-Search Confidence**	**Changed Answer, No. (%)**	**Did Not Change Answer, No. (%)**
Not confident/ somewhat confident (n = 64)	22 (34.4)	42 (65.6)
Confident/ very confident (n = 864)	51 (5.9)	813 (94.1)

**Table 9:** Changes in post-search answer before and after feedback (N = 928)

**BeforeFeedback**	**AfterFeedback**	
	**Right, No. (%)**	**Wrong, No. (%)**
Right (n= 167)	122 (73.1)	45 (26.9)
Wrong (n= 761)	14 (1.8)	747 (98.2)

## Discussion

This research demonstrates that while health care consumers can improve the accuracy of their answers to health care questions after searching quality online resources, their confidence in answers is not a good indicator of the answer being correct. Further, consumers who are not confident in their answers after searching are more likely to be influenced to change their views after feedback with other consumers’ answers.

Results of this study for nonclinically trained users are in line with studies that reported search engines can improve the ability of clinically trained users to answer questions [25,27,28]. The 21% improvement in accuracy between pre-search and post-search answers reported in this study corresponds with the study conducted by Hersh et al [28], which found that 66 medical and nurse practitioner students were able to improve their answers to a set of five clinical questions by up to 20% before and after using Medline. Our improvement rate also corresponds with the 21% improvement reported for clinicians who used the same search engine to answer eight clinical scenario questions in a controlled laboratory setting (pre-search correct 29% [95% CI: 25-33]; post-search correct 50% [95% CI: 46-54]; *z*= 9.58, *P*< .001) [25].

Findings from this research and previous studies have shown that confidence is not always a good indicator of decision accuracy [26,29]. The observation that 55.6% (95% CI: 37.3-72.4) of subjects in this study who did not know the answer before searching reported being confident or very confident in their incorrect post-search answers (DW) concurs with the result reported by Westbrook et al [26], which found that among clinicians who did not know the answer before searching and were incorrect after searching (DW), 60% of doctors and 52% of clinical nurse consultants reported being confident or very confident in their incorrect post-search answer. This has implications for large-scale national surveys (such as those conducted by the Pew Research Center), which often use confidence as a metric to infer public opinion. In addition, confidence often shapes the way people make decisions (eg, in the form of the overconfidence bias [30,31]), and studies have shown that people can experience cognitive biases while searching for online information to answer questions [32]. These biases, such as the anchoring and order effects, can influence the way people attend to and process information to make a decision. More research is needed to help users assess the impact of their levels of confidence and understand how their confidence might be shaping their beliefs and ability to attend to new information.

Our findings on the impact of social feedback also concur with studies that report people are one of the important sources of information that influence clinicians’ and health care consumers’ actions when confronted with a clinical or health-related concern [19,20,33-36]. With the role of the Internet as a social network, typified by the growing interest in sites like Wikipedia, FaceBook, and MySpace, we can envisage more consumers seeking health-related information and advice from online peer networks. However, there appears to be no prior study that has evaluated the health care impact of the social feedback that is possible through such websites. In addition, it is now clear that it is not sufficient to just provide access to reliable online resources for health care consumers. The decisions consumers make are shaped by their confidence and by the influence of their peers and broader social community. Our research suggests that connecting consumers to trustworthy and relevant networks of human resources could be a significant addition to online health resources. As consumers play an increasingly active role in managing their health, it is important not to underestimate the extent to which online search engines and online peer networks can influence the way people manage their health care.

## Abbreviations

DW: didn’t know, wrong
RR: right, right
RW: right, wrong
UNSW: University of New South Wales
WR: wrong, right
WW: wrong, wrong
